# Discrepancy between Constant Properties Model and Temperature-Dependent Material Properties for Performance Estimation of Thermoelectric Generators

**DOI:** 10.3390/e22101128

**Published:** 2020-10-04

**Authors:** Prasanna Ponnusamy, Johannes de Boor, Eckhard Müller

**Affiliations:** 1 Institute of Materials Research, German Aerospace Center (DLR), D-51170 Köln, Germany; Johannes.deboor@dlr.de; 2 Institute of Inorganic and Analytical Chemistry, Justus Liebig University Gießen, D-35392 Gießen, Germany

**Keywords:** TEG performance, device modeling, temperature profile, constant properties model, Fourier heat, Thomson heat, Joule heat

## Abstract

The efficiency of a thermoelectric (TE) generator for the conversion of thermal energy into electrical energy can be easily but roughly estimated using a constant properties model (CPM) developed by Ioffe. However, material properties are, in general, temperature (T)-dependent and the CPM yields meaningful estimates only if physically appropriate averages, i.e., spatial averages for thermal and electrical resistivities and the temperature average (TAv) for the Seebeck coefficient (α), are used. Even though the use of $\alpha_{\mathrm{TAv}}$ compensates for the absence of Thomson heat in the CPM in the overall heat balance, we find that the CPM still overestimates performance (e.g., by up to 6% for PbTe) for many materials. The deviation originates from an asymmetric distribution of internally released Joule heat to either side of the TE leg and the distribution of internally released Thomson heat between the hot and cold side. The Thomson heat distribution differs from a complete compensation of the corresponding Peltier heat balance in the CPM. Both effects are estimated quantitatively here, showing that both may reach the same order of magnitude, but which one dominates varies from case to case, depending on the specific temperature characteristics of the thermoelectric properties. The role of the Thomson heat distribution is illustrated by a discussion of the transport entropy flow based on the α(T) plot. The changes in the lateral distribution of the internal heat lead to a difference in the heat input, the optimum current and thus of the efficiency of the CPM compared to the real case, while the estimate of generated power at maximum efficiency remains less affected as it is bound to the deviation of the optimum current, which is mostly <1%. This deviation can be corrected to a large extent by estimating the lateral Thomson heat distribution and the asymmetry of the Joule heat distribution. A simple guiding rule for the former is found.

## 1. Introduction

Thermoelectric generator (TEG) materials convert a certain fraction of the heat passed through them into useful electrical power, as the charge carriers (holes/electrons) absorb the thermal energy and move from the hot side to the cold side, carrying entropy [1,2]. The transport entropy flux related to the convective heat transport is given by αj, with the Seebeck coefficient α(T) and current density *j*. Typically, a thermoelectric (TE) module consists of a series of pn leg pairs (thermocouples), electrically connected in series and thermally in parallel [3]. In steady-state conditions, the exact performance of the TEG is obtained by solving the thermoelectric heat balance equation [4] for the temperature profile T(x). In 1D, it reads

(1) $$\kappa \left(T\right)\frac{\partial^{2}T}{\partial x^{2}}+\frac{d\kappa }{dT}{\left(\frac{\partial T}{\partial x}\right)}^{2}-jT\frac{d\alpha }{dT}\frac{\partial T}{\partial x}=-\rho \left(T\right)j^{2}$$

where the thermal conductivity κ, the electrical resistivity ρ and α are the three main temperature-dependent thermoelectric properties. Here, $\frac{\partial }{\partial x}\cdot \left\{\kappa \left(T\right)\frac{\partial T}{\partial x}\right\}=\kappa \left(T\right)\frac{\partial^{2}T}{\partial x^{2}}+\frac{d\kappa }{dT}{\left(\frac{\partial T}{\partial x}\right)}^{2}$ corresponds to the (negative) divergence of the Fourier heat flux, i.e., its local change; $jT\frac{d\alpha }{dT}\frac{\partial T}{\partial x}$ corresponds to the local Thomson heat absorption driven by the change of the convective entropy flux αj related to the temperature dependence of α(T), and $\rho \left(T\right)j^{2}$ corresponds to the local Joule heat dissipation. With a typical TE material, with κ(T) falling with T and the amount of α(T) rising with T, Thomson heat will be released and Fourier heat flow will grow from the hot to the cold side along a TE leg in TEG operation, where the current flow is driven by the thermo-voltage generated by the leg. Equation (1) is a second-order non-linear partial differential equation, which can be solved using numerical methods like finite element methods (FEMs) [5,6], finite volume methods (FVMs) [7,8,9] or finite difference methods (FDMs) [6]. However, these solution methods are costly and time-consuming.

On the other hand, when assuming constant properties of the TE properties, an approximate solution can be found analytically, as suggested by Ioffe [1]. This solution by the constant property model (CPM) involves a discrepancy from the exact results due to the underlying simplification. Moreover, the choice of the averaged constant properties to be obtained from the actual temperature-dependent data is not straightforward. As can be seen from Equation (1), the Thomson heat vanishes when the Seebeck coefficient (and with that the convective entropy flux αj) remains constant. Various models corrected the CPM to compensate for this “missing Thomson heat” [10,11,12,13,14,15,16] have been proposed. Meanwhile, Sandoz et al. [17] attempted to explain the use of the T-averaged Seebeck coefficient in predicting exact power in the CPM mathematically, but did not recognize the importance of the asymmetry in heat distribution for the prediction of efficiency.

In a previous study [18], on the physically appropriate choice of averages in the CPM, we highlighted that spatial averages (SpAv) for resistivities (electrical and thermal) and temperature averaging (TAv) for the Seebeck coefficient are essential for a meaningful CPM estimate. However, there is still a remaining deviation due to unconsidered local redistribution of internal heat release or absorption and of thermal conduction in the CPM, which is linked to a change in the *T* profile *T*(*x*) [1,12,18,19]. Here, we will analyze the individual heat contributions exemplarily for six representative thermoelectric materials that we considered previously [18] plus PbTe [20], as this is one of the best TE materials in practice and shows an especially large deviation between CPM and exact results.

Initially, the effect of the *T* dependence of each of the TE properties, α, ρ and κ, leading to locally shifted heat release and transport over the TE element, for performance estimation, is studied separately. Calculated maximum efficiency in the full temperature-dependent case, $\eta_{\max}$, is compared with tailored model materials, in order to separate and quantify the individual contributions. Model materials are defined by setting one or two of the three TE properties constant at its respective average while keeping the other properties T dependent. Next, we explain the physical origin of a relevant part of the discrepancy between CPM results and the real situation using a schematic plot of the convective entropy flux derived from an α(T) graph, alongside showing that the net Peltier/Thomson heat is correctly considered by the CPM when appropriate temperature averaging is used for α(T). Marked areas in the entropy flux diagram quantify the exchange of Peltier and Thomson heat, and with that, a correction for the related deviation in CPM efficiency estimation, $d\eta_{\max}=\frac{\eta_{\max}-\eta_{\max}{}^{\mathrm{CPM}}}{\eta_{\max}{}^{\mathrm{CPM}}}$, is suggested and demonstrated.

## 2. Methods, Results and Discussion

### 2.1. Role of the T Dependence of Material Properties in Performance Estimation

Since a generalized temperature dependence study for all types of T dependence is quite elaborate, a comparative study based on seven well-known and representative TE materials [20,21,22,23,24] was conducted. To understand the role of the T dependence of each of α, ρ and κ in performance estimation, the calculated maximum efficiencies when all properties are considered as T dependent (referred to as “real case” or “exact” from now on) were compared with the calculated efficiencies of model materials. These model materials have the same T dependence as the real materials for one or two of the three thermoelectric transport properties, while the remaining properties are kept constant; these materials are denoted as two temperature-dependent property (2TD) materials and 1TD materials, respectively. The constants used to define the model materials were obtained using the spatial averages (SpAv; for electrical and thermal resistivity) at a current density corresponding to the maximum efficiency of the real material and the temperature average (TAv; for the Seebeck coefficient). The SPAv and TAv of a T-dependent quantity *p* for a hot side temperature $T_{h}$ and a cold side temperature $T_{c}$ are given by [1,12,18,25]

(2) $$p_{\mathrm{TAv}}=\bar{p}=\frac{1}{\Delta T}\int_{T_{c}}^{T_{h}}p\left(T\right)dT$$

(3) $$p_{\mathrm{SpAv}}=\langle p\rangle =\frac{1}{L}\int_{0}^{L}p\left(T\left(x\right)\right)dx$$

where $\Delta T=T_{h}-T_{c}$ and L are the length of the TE leg. The exact efficiency using T-dependent properties was obtained using the 1D solution algorithm developed in [18] by calculating

(4) $$P=V\cdot I,\;\mathrm{where}\;V=V_{o}-R_{\mathrm{in}}I,V_{o}=\alpha \Delta T\;\mathrm{and}$$

(5) $$\eta =P/Q_{\mathrm{in}}$$

Here, P is the output power, V is the net output voltage which is given by the Seebeck voltage generated, $V_{o}=\int_{T_{c}}^{T_{h}}\alpha \left(T\right)dT,$ minus the voltage drop due to internal resistance $R_{i}=\frac{\rho_{\mathrm{SpAv}}L}{A},\;$ where A is the area of the TE leg and $\rho_{\mathrm{SpAv}}=\frac{1}{L}\int_{0}^{L}\rho \left(T\left(x\right)\right)dx$. I=jA is the current passing through the TE material due to the generated voltage. The efficiency (η) is given by the ratio of output power to the input heat flow ($Q_{\mathrm{in}}$) as in Equation (5), where $Q_{\mathrm{in}\;}$ is given by

(6) $$Q_{\mathrm{in}}=-\kappa_{h}\cdot A\cdot {\frac{dT}{dx}}_{h\;}+I\cdot \alpha_{h}\cdot T_{h}$$

$Q_{\mathrm{in}}$ consists of the Fourier heat flow $-\kappa_{h}\cdot A\cdot {\frac{dT}{dx}}_{h}$ (including the fraction of Joule and Thomson heat contributions released in the leg which is flowing to the hot side) plus the Peltier heat ($I\cdot \alpha_{h}$*∙* $T_{h})$ absorbed at the hot side. The suffix h indicates the hot side values, i.e., $\kappa_{h}=\kappa \left(T_{h}\right)$ and $\alpha_{h}=\alpha \left(T_{h}\right)$. As the spatial averages depend on T(x), which in turn varies with current, they were formed pre-assuming the optimum current of the real materials. For brevity, the efficiency was also calculated at the optimum current of the real material. The optimum current in the numerical calculation was obtained by finding the current where $\frac{d\eta }{dI}$ becomes zero.

**Figure 1:**
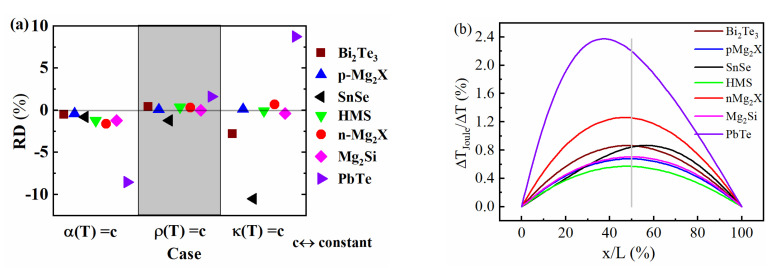
(**a**) Comparison of the relative deviation of the calculated maximum efficiency of 2TD (two temperature-dependent property) model materials (one of the thermoelectric properties kept constant,) to their real counterpart for the example materials, (**b**) T profile bending caused by Joule heat for example materials. Distinct asymmetry is observed particularly for PbTe and SnSe, correlated to maximum offset values in the middle part of Figure (**a**).

The relative deviation (RD) of the calculated maximum efficiency between the 2TD model materials and the real materials, $\delta \eta_{\max}^{\mathrm{model}}=\frac{\eta_{\max}-\eta_{\max}{}^{\mathrm{model}}}{\eta_{\max}{}^{}}$, is shown in Figure 1a. Here, and in the following, for brevity, we will use δ and d to denote a relative and absolute deviation, respectively. The comparison shows how strongly each of the contributing *T* dependences alone would shift efficiency. Obviously, the *T* dependence of ρ will affect the calculated efficiency to a lower extent than α(T) and κ(T) will do for some materials (middle section of Figure 1a); the asymmetry of Joule heat generation mostly plays a minor role. However, this does not hold for all materials and it does not mean that the RD between the CPM and a real material due to asymmetric distribution of Joule heat, $\delta \eta_{\max}{}_{J}=\frac{d\eta_{\max}}{d{\dot{Q}}_{J}^{h}}\delta {\dot{Q}}_{J}^{h}$, would be insignificant, as all of the three identified effects will act simultaneously when comparing the CPM and the real case. Although the effects of the *T* dependence of α(T) and κ(T) are much larger for some materials, they often partly cancel each other. A comparison of the real Joule heat partial T profiles in Figure 1b shows a considerable asymmetry, in correlation to the deviations in the ρ(T)=const. case for SnSe and PbTe (Figure 1a, mid); however, the RD contribution related to the profiles in Figure 1b is larger as they contain an asymmetry due to the asymmetry of axial heat conduction linked to κ(T), in addition to the asymmetry of Joule heat generation which alone is represented by Figure 1a. Calculation of the partial *T* profiles is explained in Appendix A.2. It should be noted that unlike for α(T), where the absence of the *T* dependence means an absence of Thomson heat, the absence of the *T* dependence of ρ just means that there is no local asymmetry in Joule heat generation, whereas the amount of Joule heat that appears remains unchanged. Both symmetrically or asymmetrically released Joule heat will contribute, together with Thomson heat, to the effect of a *T* dependence of κ(T) that consists in shifting the distribution of the inner reversible and irreversible heat towards the hot and cold sides. Accordingly, the magnitude of the effect of a *T* dependence of κ(T) will scale with the total amount of inner heat.

**Figure 2:**
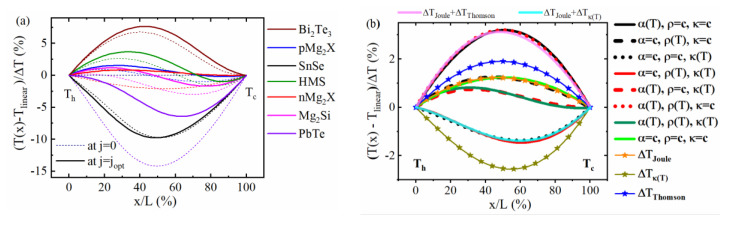
(**a**) Bending of T profiles for the real materials at j=0 (dotted lines) and $j=j_{\mathrm{opt}}$ (solid lines), normalized to ΔT, (**b**) T profile bending for the 1TD and 2TD model materials in comparison to the full T -dependent case and the constant properties case, along with the individual contributions to the fully T-dependent profile for an n-Mg_2_(Si,Sn) TE leg with $T_{h}$ = 723 K and $T_{c}$ = 383 K.

**Figure A1:**
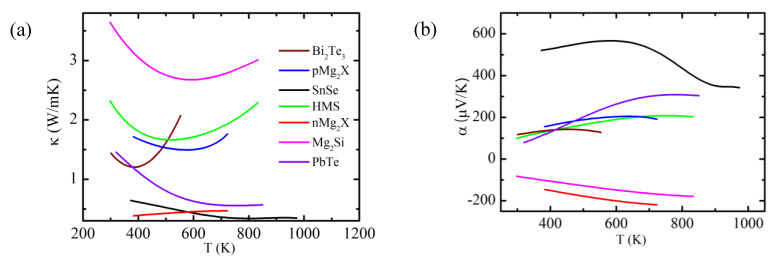

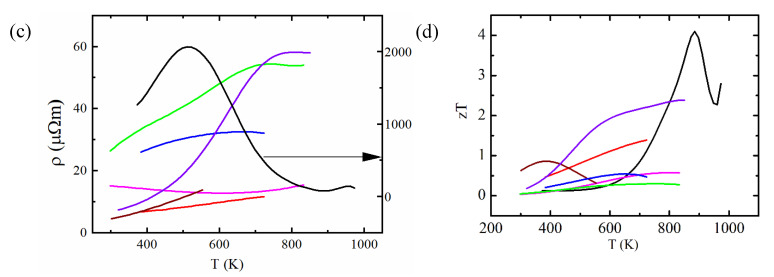
Temperature-dependent thermoelectric material properties of representative material classes: (**a**) thermal conductivity, (**b**) Seebeck coefficient, (**c**) electrical resistivity and (**d**) figure of merit. Since SnSe has much higher resistivity, the scale for it is given on the right y-axis. All the raw experimental data taken from the literature [20,21,22,23,24] were fitted with appropriate polynomials (usually 3rd or 4th order). For SnSe, a 9th order polynomial fit was used owing to the complex T dependence and hence shows an unusually high zT_max_. However, this does not affect the physics discussed and hence these fitted data were used throughout the manuscript.

**Figure A2:**
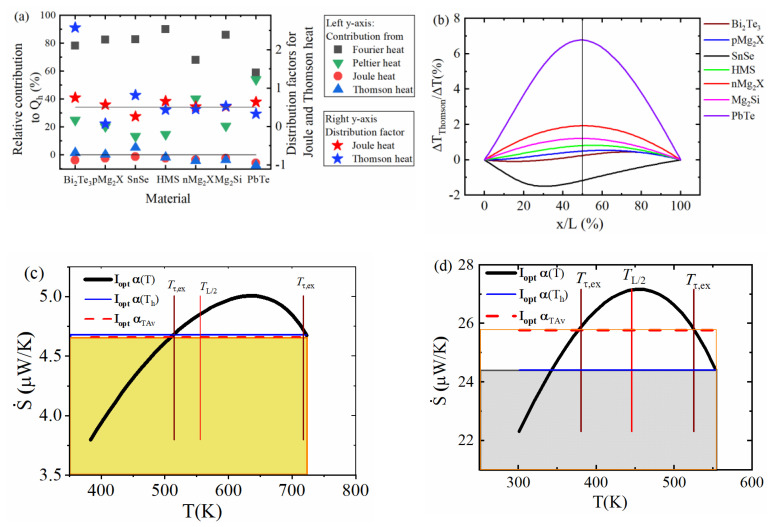
(**a**) Ratio of individual heat contributions to ${\dot{Q}}_{\mathrm{in}}\;$(Equation (A1)) calculated from the corresponding partial temperature profiles (for comparison, all quantities are counted as positive when flowing into the element) (left *y*-axis), and distribution factors (right *y*-axis) for Thomson and Joule heat. (**b**) Thomson T profiles for all example materials (**c**) $\dot{S}\left(T\right)$ diagram for p-Mg2X showing the area between $I\alpha \left(T_{h}\right)$ and $I\alpha_{\mathrm{TAv}}$ (corresponding to the Peltier heat difference between the CPM and real case), which is very small due to the shape of α(T). The position of the first peak in the Thomson partial T profile is marked as a brown vertical line. (**d**) $\dot{S}\left(T\right)$ diagram for Bi_2_Te_3_,where $\alpha_{\mathrm{TAv}}>\alpha \left(T_{h}\right)$. Hence, ${\dot{Q}}_{\pi ,h}^{\mathrm{CPM}}$ is higher than ${\dot{Q}}_{\pi ,h}$.

When α or κ is kept constant, there can be large discrepancies, as seen from the scatter in the left and right section of Figure 1a. Switching off Thomson heat results in a change from non-constant to constant convective entropy flux linked to a different partition of reversible (Peltier + Thomson-bound) heat to both sides of the leg. When setting κ(T)=const., net Fourier heat transmitted does not change as the thermal resistance of the leg is fixed by the definition of the SpAv. Rather, the observed differences are merely due to a changed lateral distribution of Thomson and Joule heat. Comparing this to Figure 2a reveals that a large RD for κ(T)=const. correlates to strongly non-linear *T* profiles linked to κ(T) (see T profiles for j = 0); see also Appendix A.2, Figure A1a, where SnSe, Bi_2_Te_3_ and PbTe have significantly different $\kappa_{h}$ and $\kappa_{c}$ and Figure A2a, showing that the weight of Joule and Thomson heat to $Q_{\mathrm{in}}$ is comparably large for these materials.

The dominating effect of the *T* dependence of κ and α on the estimated performance is also seen by comparing the *T* profiles of the model cases with the real temperature profile of *n*-type Mg_2_(Si,Sn) (referred to as n-Mg_2_*X*), Figure 2b. All profiles are calculated for the optimum current for maximum efficiency of the real material. Here, in addition to the 2TD materials, 1TD materials were also involved. α(T) and κ(T) play a dominating role in the shaping of the temperature profile, which is reflected by the closeness of the α(T)≠const., κ(T)≠const. case to the real material.

The effects of the 2TD cases on the overall inflowing Fourier heat and thus on the efficiency of n-Mg_2_*X* from Figure 1a (red dots) can be discussed in terms of the hot side slopes of the corresponding temperature profiles (red lines) in Figure 2b when comparing between cases with the same κ(T). The downward ${\frac{dT}{dx}}_{h\;}$ for the 2TD material with α(T)=const.(red solid line) indicates an increase in the inflowing Fourier due to missing Thomson heat, compared to the actual case (dark green line). Simultaneously, but only partly compensated in the $Q_{\mathrm{in}}$ balance by missing Thomson heat, less Peltier heat is absorbed at the hot side and therefore the efficiency is overestimated (Figure 1a left side, red dot). The 2TD κ(T)=const.(red dotted line) deforms the *T* profile considerably but hardly increases the heat input (Equation (6)) compared to the real material, as the SpAv of κ(T) maintains an unchanged thermal resistance of the TE leg. We can conclude that replacing the T dependence of α(T) and κ(T) by adequate constants will, although significantly changing the T profile, influence the inflowing heat and thus efficiency to a much lower extent due to compensating effects. The RD of CPM efficiency in effect arises mainly from a redistribution of internal Joule and Thomson heat due to considerable deformation of the *T* profile by neglecting the *T* dependence of κ(T) and α(T) and local redistribution of reversible heat generation as a consequence of neglect of the *T* dependence of the convective entropy flux.

When comparing the 1TD and 2TD model materials, additionally a shift of the SpAv values of ρ and κ as a consequence of different T profiles, as well as coupling effects among the individual contributions, play a role, but only to a very minor extent, as proven by the close coincidence of their profiles to combinations of the individual partial *T* profiles of the real material, see Figure 2b (pink and cyan lines). The latter represent the physical contributions to the real temperature profile, $\Delta T_{\mathrm{Joule}},\;\Delta T_{\mathrm{Thomson}}$ and $\Delta T_{\kappa \left(T\right)}$ and are plotted by symbols and lines in Figure 2b. They sum up, together with the linear part, $T_{\mathrm{lin}}\left(x\right)=T_{h}-x\frac{\Delta T}{L}$, to the total temperature profile

(7) $$T\left(x\right)=T_{\mathrm{lin}}\left(x\right)+\Delta T_{\mathrm{Joule}}\left(x\right)+\Delta T_{\mathrm{Thomson}}\left(x\right)+\Delta T_{\kappa \left(T\right)}\left(x\right)$$

The procedure to calculate the partial profiles is described in Appendix A.3.

From the close coincidence of combinations of the real partial *T* profiles to the *T* profiles of the 1TD and 2TD model materials, as evident from Figure 2b, we can conclude that the contributions from each of the effects (Thomson heat, Joule heat, *T* dependence of κ) to the total T(x) behave in good approximation and are independent and additive (a small note on this is given in the Appendix A.1.). The reason for the overall weak cross-coupling between the contributing effects is the small amplitude of the partial T profiles $\Delta T_{\mathrm{Joule}}$, $\Delta T_{\mathrm{Thomson}}$, $\Delta T_{\kappa \left(T\right)}$ compared to the overall ΔT but also the fact that $\Delta T_{\mathrm{Thomson}}$ and $\Delta T_{\kappa \left(T\right)}$ often partially compensate. Therefore, the T profiles of a real material and the CPM may also be quite close to each other for some materials. It is evident that the shape of α(T) and κ(T) affects the temperature profile much more than that of ρ(T) but this does not mean that the asymmetry of Joule heat distribution between the hot and cold side would contribute insignificantly to the difference of the inflowing heat between the CPM case and a real material. The redistribution of Joule heat affects the maximum efficiency to a relevant extent along with the redistribution of Thomson heat. Thus, we can split the RD of the maximum efficiency according to the physical origin—redistribution of Peltier–Thomson heat and Joule heat—as $\delta \eta_{\max}=\frac{\eta_{\max}-\eta_{\max}{}^{\mathrm{CPM}}}{\eta_{\max}{}^{\mathrm{CPM}}}=\delta \eta_{\max}{}_{\pi \tau }+\delta \eta_{\max}{}_{J}$.

Depending on the slope ratio of κ(T) and α(T), the efficiency discrepancy due to Joule heat asymmetry, $\delta \eta_{\max}{}_{J}$, will vary considerably between different materials and may change sign from case to case, as observed in [18].

Now, let us proceed to understand in more detail how the absence of Thomson heat in the CPM will affect the efficiency calculation. We will see that it is partially and usually not entirely compensated by the difference in Peltier heat between a real material and its CPM approximation.

### 2.2. Peltier–Thomson Heat Balance and the Resulting Uncertainty in CPM Efficiency

**Figure 3:**
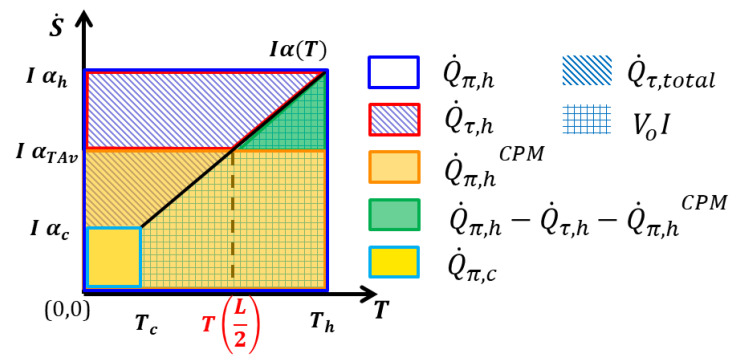
Schematic representation of reversible heat exchange in a TE leg for a linear α(T) curve (black line) in a plot of a convective 1D entropy flow with a constant current I. According to the relation ${\dot{Q}}_{\pi }=I\alpha T$, areas in the $\dot{S}\left(T\right)$ diagram represent certain amounts of (flowing or exchanged) Peltier (including Thomson) heat. The dark blue and light blue rectangles—in- and outflowing Peltier heat; trapezium above the $\dot{S}\left(T\right)$ curve—Thomson heat (marked with slant lines); trapezium below the $\dot{S}\left(T\right)$ curve (marked in checked lines) —gross electrical power generated ($V_{o}I)$; red trapezium—Thomson heat flowing to the hot side; orange rectangle—hot side Peltier heat (CPM). The green triangle indicates part of the difference in the amount of absorbed Peltier heat at the hot side between the actual and the CPM cases that is not compensated in the real material by backflowing Thomson heat ${\dot{Q}}_{\tau ,h}$.

Consider a TE material with constant κ and a linearly increasing α(T) curve (which is typical for a TE material below the peak *zT* temperature), as schematically shown in Figure 3. In a TE material under current flow, the convective entropy flux is given by $\dot{s}\left(T\right)=j\alpha \left(T\right)$. Hence, in a TE leg with a current flow *I*, the convective entropy flow $\dot{S}\left(T\right)=I\alpha \left(T\right)$ is directly linked to the temperature dependence of the Seebeck coefficient.

Peltier heat absorbed at the hot side $(T_{h}$) in the real case is given by ${\dot{Q}}_{\pi ,h}$ *=* $I\alpha_{h}T_{h}$*,* while at the cold side, it is ${\dot{Q}}_{\pi ,c}$*=* $I\alpha_{c}T_{c}$. Areas in the diagram of Figure 3 represent certain amounts of Peltier and Thomson heat but also generated electric power. This allows a schematic comparison of reversible heat exchange in a T-dependent material to its CPM approximation. The difference in the Peltier heat balance, $I\left(\alpha_{h}T_{h}-\alpha_{c}T_{c}\right)$, is given by the difference of the light and dark blue line-marked areas. It is composed of the area below the $\dot{S}\left(T\right)$ curve (marked in checked lines) given by $P_{0}=IV_{0}=I\int_{T_{c}}^{T_{h}}\alpha \left(T\right)dT$, which is the gross produced electrical power (which includes Joule heat). The area to the left from the $\dot{S}\left(T\right)\;$ curve (indicated by slant lines) is

(8) $$\int_{I\alpha_{c}}^{I\alpha_{h}}Td\dot{S}=I\int_{T_{c}}^{T_{h}}T\frac{d\alpha }{dT}dT=I\int_{T_{c}}^{T_{h}}\tau dT={\dot{Q}}_{\tau }$$

where $\tau =T\frac{d\alpha }{dT}$ is the Thomson coefficient. This area represents the net Thomson heat generated in the TE leg, ${\dot{Q}}_{\tau }$, which is directly linked to the variation of the convective entropy flow over the leg. The reversible heat balance

(9) $${\dot{Q}}_{\pi ,h}-\;{\dot{Q}}_{\pi ,c}={\dot{Q}}_{\tau }+P_{0}$$

shows that the loss of Peltier heat in the sample equals released Thomson heat plus produced gross electrical power. ${\dot{Q}}_{\tau }$ and $P_{0}$ are counted here as positive when going out of the system. Part of the Thomson heat will flow back, as a contribution to the overall Fourier heat flow, to the hot side. For simplification we assume that Thomson heat that is released at any point in the leg will flow out to the closer side. This is physically not strict but sufficient to qualitatively illustrate the relevant effect of undercompensation of the difference in Peltier heat exchanged at the hot side in a real material compared to the CPM by Thomson heat flowing back to the hot side, i.e., compensation of $d{\dot{Q}}_{\pi ,h}={\dot{Q}}_{\pi ,h}-{\dot{Q}}_{\pi ,c}{}^{\mathrm{CPM}}=IT_{h}\left(\alpha_{h}-\bar{\alpha }\right)$ by ${\dot{Q}}_{\tau ,h}=I\int_{\alpha_{\tau ,\mathrm{ex}}}^{\alpha_{h}}Td\alpha$. The relevant question on the Seebeck value $\alpha_{\tau ,\mathrm{ex}}$, from which the integration gives the correct amount of ${\dot{Q}}_{\tau ,h}$ (and its corresponding temperature $T_{\tau ,\mathrm{ex}}$ with $\alpha_{\tau ,\mathrm{ex}}=\alpha \left(T_{\tau ,\mathrm{ex}}\right)$), will be touched on below.

In the CPM, the Peltier heat at the hot side is given by $I\bar{\alpha }T_{h}$, while at the cold side it is $I\bar{\alpha }T_{c}$, where $\bar{\alpha }=\alpha_{\mathrm{TAv}}$ is the temperature average of α(T) (see Equation (2)). Therefore, the following equation holds:

(10) $${\dot{Q}}_{\pi ,h}{}^{\mathrm{CPM}}-{\dot{Q}}_{\pi ,c}{}^{\mathrm{CPM}}=\;I\bar{\alpha }(T_{h}-T_{c})=I\int_{T_{c}}^{T_{h}}\alpha \left(T\right)dT$$

i.e., Peltier heat is completely balanced by electrical production.

From Equations (9) and (10), it is obvious that globally the explicit absence of Thomson heat in the CPM is taken care of correctly by the use of temperature averaged $\bar{\alpha }$ in the CPM, i.e.,

(11) $${\dot{Q}}_{\pi ,h}-{\dot{Q}}_{\pi ,c}-{\dot{Q}}_{\tau }={\dot{Q}}_{\pi ,h}{}^{\mathrm{CPM}}-{\dot{Q}}_{\pi ,c}{}^{\mathrm{CPM}}=I\bar{\alpha }\Delta T=P_{0}$$

With this choice of $\bar{\alpha }\;$ as the CPM value, the gross power generated is exactly the same in the CPM as in the real material, at the same current. On the other hand, it implies that, typically, considerably less Peltier heat is absorbed at the hot side in the CPM case than in reality, whereas back-flowing Thomson heat partly compensates the actually higher Peltier heat intake. Figure 3 visualizes with the green triangle that this compensation is incomplete, i.e., $d{\dot{Q}}_{\pi \tau ,h}=d{\dot{Q}}_{\pi ,h}-{\dot{Q}}_{\tau ,h}>0$. Accordingly, more Thomson heat is leaving at the cold side. It is evident that this holds not only for a linear but also for a left- or right-hand bowed Seebeck curve.

**Figure 4:**
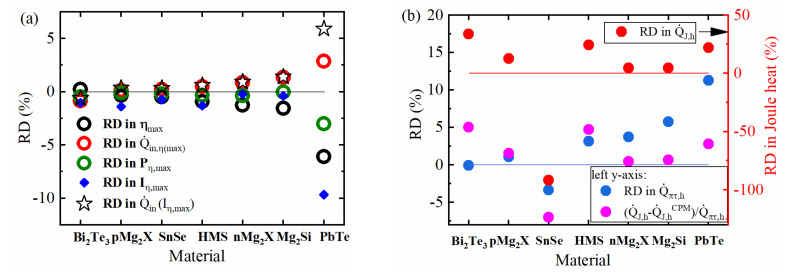
Calculated relative deviation (RD) of (**a**) the maximum efficiency, $\delta \eta_{\max}$, heat input, $\delta {\dot{Q}}_{\mathrm{in}}$, power at maximum efficiency, $\delta P_{\eta_{\max}}$, and optimum current, $\delta I_{\mathrm{opt},\eta }$; additionally, $\delta {\dot{Q}}_{\mathrm{in}}$ when neglecting $\delta I_{\mathrm{opt},\eta }$ (black stars), (**b**) Joule heat, $\delta {\dot{Q}}_{J}^{h}$, reversible heat, $\delta {\dot{Q}}_{\pi \tau }^{h}$, (see Equation (12)) and, for direct comparison, also $d{\dot{Q}}_{J}^{h}/{\dot{Q}}_{\pi \tau }^{h}$.

**Figure 5:**
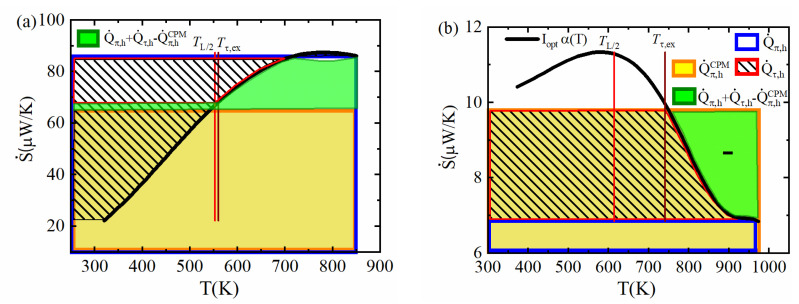
Plot of the convective 1D entropy flow at constant current I for (**a**) PbTe and (**b**) SnSe. Relevant areas are marked to determine the uncompensated Peltier–Thomson heat $d{\dot{Q}}_{\pi \tau }^{h}$ (green area). Note that the $\frac{L}{2}$ temperature and the temperature $T_{\tau ,\mathrm{ex}}$ according to the extremum of $\Delta T_{\mathrm{Thomson}}\left(x\right)$ may be located quite far apart (**b**) whereas $T_{\tau ,\mathrm{ex}}$ is very close to the crossing point of α(T) to $\bar{\alpha }$.

**Figure 6:**
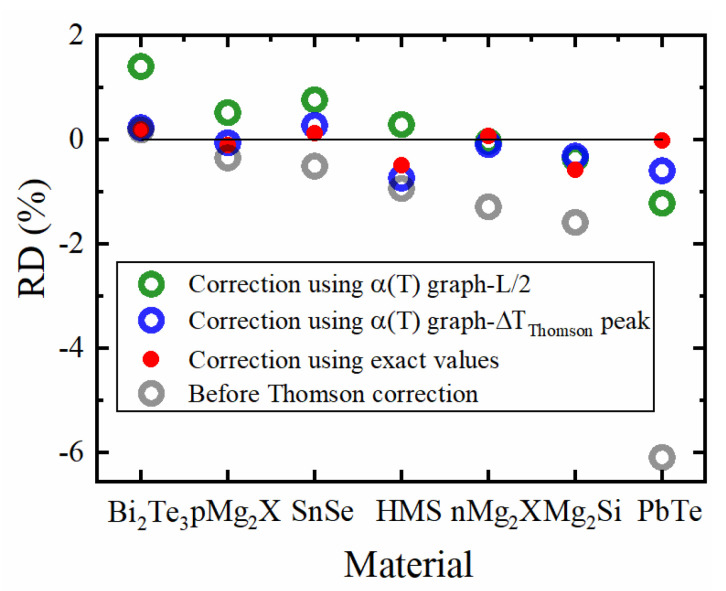
RD in maximum efficiency, $\delta \eta_{\max}^{\mathrm{corr}}$, corrected with respect to ${d\dot{Q}}_{\pi \tau }^{h,\;I=\mathrm{const}}$ ($T_{\tau ,\mathrm{ex}}$ according to the peak of the exact Thomson profile; blue), ${d\dot{Q}}_{\pi \tau }^{h}$ (exact numerical calculation; red; compare also Equation (12)) and a first guess by the $\frac{L}{2}$ position.

In a less typical case with strongly asymmetric heat conduction, i.e., κ(T) strongly increasing with T, or if α(T) forms a significant maximum, this typical tendency could reverse, but mostly it leads to underestimation of the inflowing heat in the CPM case $Q_{\mathrm{in}}{}^{\mathrm{CPM}}$ and hence to overestimation of the efficiency by the CPM. With p-Mg_2_*X*, a particular example is given in Appendix A.3.2 (Figure A2c) where, with α(T) weakly changing between $T_{c}$ and $T_{h}$ but peaking inside, this compensation can also be almost perfect, or, as for SnSe (Figure 4, Figure 5b and Figure 6), overcompensation may even occur.

Overall, the efficiency deviation between the real and CPM cases would be negligible if $Q_{\mathrm{in}}=Q_{\mathrm{in}}{}^{\mathrm{CPM}}$. For a rising α(T) curve, which is the typical case applied for most of the established TE materials, the Peltier–Thomson part, ${\dot{Q}}_{\pi \tau }^{h}{}^{\mathrm{CPM}}$, of ${\dot{Q}}_{\mathrm{in}}$ will remain lower than the real ${\dot{Q}}_{\pi \tau }^{h}$. Thus, the efficiency is often overestimated by the CPM. Furthermore, a shift in $I_{\eta ,\mathrm{opt}}{}^{\mathrm{CPM}}$ against the true $I_{\eta ,\mathrm{opt}}$ has to be taken into consideration due to a change in the current-dependent contributions to ${\dot{Q}}_{\mathrm{in}}$. The usually higher intake of reversible heat at the hot side in the real case, ${\dot{Q}}_{\pi \tau }^{h}$, compared to the CPM ($\delta {\dot{Q}}_{\pi \tau ,h}>0$) results in a steeper curve ${\dot{Q}}_{\mathrm{in}}\left(I\right)$ than ${\dot{Q}}_{\mathrm{in}}{}^{\mathrm{CPM}}\left(I\right)$. Efficiency, as defined by $\eta \left(I\right)=P/{\dot{Q}}_{\mathrm{in}}$, will accordingly have a lower slope in reality than for the CPM, equivalent to a lower maximum position $I_{\mathrm{opt},\eta }$. Thus, usually, the CPM will overestimate the optimum current, $\delta I_{\mathrm{opt},\eta }=\frac{I_{\mathrm{opt},\eta }-I_{\mathrm{opt},\eta }^{\mathrm{CPM}}}{I_{\mathrm{opt},\eta }^{\mathrm{CPM}}}<0$, and hence will overestimate output power at maximum efficiency ($\delta P_{\eta_{\max}}^{}<0$), which adds to the overestimate of maximum efficiency: $\delta \eta_{\max}=\delta P_{\eta_{\max}}^{}-\delta {\dot{Q}}_{\mathrm{in}}$, amplifying the effect of $\delta {\dot{Q}}_{\mathrm{in}}$ (see Figure 4a). Hence, for a quantitative analysis, we have to consider three contributions to the (absolute) deviation of ${\dot{Q}}_{\mathrm{in}}$

(12) $${d\dot{Q}}_{\mathrm{in}}={d\dot{Q}}_{\pi \tau }^{h}-{d\dot{Q}}_{J}^{h}={\;d\dot{Q}}_{\pi \tau }^{h,\;I=\mathrm{const}}-{d\;\dot{Q}}_{J}^{h,\;I=\mathrm{const}}+\;{\frac{\partial {\dot{Q}}_{\mathrm{in}}}{\partial I}|}_{I_{\mathrm{opt},\eta }}{\mathrm{dI}}_{\mathrm{opt},\eta }$$

where, similar to the outflowing Thomson heat, outflowing Joule heat is also counted as positive and ${d\dot{Q}}_{J}^{h}$ is due to the Joule heat asymmetry at the hot side. Asymmetry of Joule heat distribution and heat conduction will, with falling κ(T), as for PbTe and SnSe, favor heat release to the cold side. This will likewise contribute to a higher ${\dot{Q}}_{\mathrm{in}}$ and steeper ${\dot{Q}}_{\mathrm{in}}\left(I\right)$, amplifying the same trend as for reversible heat, or will counteract it with rising κ(T). Thus, asymmetry of Joule heat distribution will add to the mispoint in $I_{\mathrm{opt},\eta }{}^{\mathrm{CPM}}$.

Figure 4a shows that for most materials, $I_{\mathrm{opt},\eta }$ changes for about 1% or less and, consequently, also the deviation of the output power, remain small. However, for PbTe, $\delta I_{\mathrm{opt},\eta }$ reaches 10%. Then the deviation of output power, $\delta P_{\eta_{\max}}$, may grow in absolute amount to be as large as $\delta {\dot{Q}}_{\mathrm{in}}$, doubling its effect. Whereas the contribution to $\delta {\dot{Q}}_{\mathrm{in}}$, due to $\delta I_{\mathrm{opt},\eta }$ usually remains insignificant, it becomes relevant for PbTe where it compensates half of $d{\dot{Q}}_{\mathrm{in}}$ related to the distribution of inner heat at an unchanged current, ${d\dot{Q}}_{\pi \tau }^{h}\left(I_{\mathrm{opt},\eta }\right)-{d\dot{Q}}_{J}^{h}\left(I_{\mathrm{opt},\eta }\right)$, see Equation (12) and black stars in Figure 4a.

The RD of hot side Joule heat, $\delta {\dot{Q}}_{J}^{h}$, and Peltier/Thomson heat, $\delta {\dot{Q}}_{\pi \tau }^{h}$ with ${\dot{Q}}_{\pi \tau }^{h}={\dot{Q}}_{\pi }^{h}-{\dot{Q}}_{\tau }^{h}$, are shown in Figure 4b. $\delta {\dot{Q}}_{J}^{h}$ reaches quite significant nominal values (SnSe), mainly due to the low magnitude of ${\dot{Q}}_{J}^{h}$ itself. For direct comparison to $\delta {\dot{Q}}_{\pi \tau }^{h}$, the (absolute) deviation $d{\dot{Q}}_{J}^{h}$ related to ${\dot{Q}}_{\pi \tau }^{h}$ is plotted and shows that both effects reach the same order of magnitude. Typically, both contributions partly compensate. Furthermore, no general behavior can be observed in their mutual relation over the materials, as in some cases clearly one effect dominates, in others the other.

As seen from Figure 1b, usually, more Joule heat is released to the hot side than to the cold side in a real material, whereas there are symmetric amounts in the CPM case. This contributes to an underestimation of the efficiency in the CPM case, $\delta \eta_{\max}{}_{J}>0$. On the other hand, as explained, the Peltier–Thomson balance tends to an overestimation, $\delta \eta_{\max}{}_{\pi \tau }<0$, thus, both effects counteract and partially compensate. From Figure 4a, it can be seen that the CPM overestimates the efficiency compared to the real case for all selected materials except Bi_2_Te_3_, which has an exceptionally higher $\kappa_{h}$ compared to the cold side (Figure A1a in Appendix A.2) together with high Joule release (Figure 4b) and almost compensation of the Peltier–Thomson balance. Thus, the Joule contribution dominates, leading to an underestimation of the efficiency. Additionally, SnSe behaves somewhat differently from the general trend, with a falling α(T) curve (Appendix A.2 Figure A1b) and the over-resistivity at the cold side (Appendix A.2 Figure A1c). Moreover, $\kappa_{h}$ is much lower than $\kappa_{c}$. As an effect, Joule heat is preferentially led to the cold side; consequently, hot side Joule heat is greatly overestimated in the CPM (Figure 4b), but as the relative contribution of Joule heat to $Q_{\mathrm{in}}$ is small (Figure A2a), the resulting trend towards the overestimation of performance in the CPM remains moderate. On the other hand, as seen from Appendix A.3.2 Figure A1b, Thomson heat is absorbed in the leg as α(T) for SnSe is a falling curve and is mainly bound to the hot side. As seen from Figure 4b, for SnSe, the hot side Peltier–Thomson heat will, unlike for most of the other materials, be overestimated by the CPM. However, the resulting underestimation of efficiency in the CPM will be overcompensated by the counteracting Joule heat distribution.

The first four materials in our list (see Figure 4a) show a minor discrepancy of the CPM with reality. Although Joule heat asymmetry is contributing comparably, from case to case, the dominating source of discrepancy is mostly the uncompensated Peltier heat according to Equation (11)). It is particularly relevant in the cases of n-Mg_2_*X*, Mg_2_Si and PbTe, which have larger Thomson contributions (Figure A2a), leading to larger discrepancies of the CPM efficiency estimate.

### 2.3. Refining the CPM Efficiency Estimate

Having identified the effects causing a systematic uncertainty in the CPM efficiency estimation, they can be accordingly corrected.

We want to analyze how this can be done practically for the Thomson contribution, $\delta {\dot{Q}}_{\pi \tau }^{h}$, by calculating the uncompensated Peltier heat at the hot side. Therefore, we discuss the approach for example materials with dissimilar α(T) characteristics.

The values of ${\dot{Q}}_{\pi ,h}$ and ${\dot{Q}}_{\pi ,h}^{\mathrm{CPM}}$ are known from $T_{h}$, $\alpha_{h}$ and $\bar{\alpha }$, for a given current, where, as a first approximation, $I_{\mathrm{opt},\eta }^{\mathrm{CPM}}$ is used. We have seen that the Thomson heat flowing to the hot side is strictly calculated from the partial *T* profile $\Delta T_{\mathrm{Thomson}}\left(x\right)$ by ${\dot{Q}}_{\tau ,h}=-\kappa_{h}\cdot {\frac{d\Delta T_{\mathrm{Thomson}}}{dx}}_{h\;}$. We apply this route to form a reference for an approximate estimation to be developed and, because of this, we omit a numerical calculation of exact *T* profiles. As derived from Equation (10), we obtain the uncompensated Peltier–Thomson heat from $d{\dot{Q}}_{\pi \tau }^{h}={\dot{Q}}_{\pi ,h}+{\dot{Q}}_{\tau ,h}-{\dot{Q}}_{\pi ,h}{}^{\mathrm{CPM}}$. Neglecting any deviation of current, this can be illustrated in the α(T) diagram based on our interpretation of areas by amounts of reversible heat, see Figure 3. Thus, we aim for a good approximation of the green marked area in Figure 3 by an appropriateand simple approximation. The problem splits into two aspects: finding the temperature $T_{\tau ,\mathrm{ex}}$ above which the inner Thomson heat is conducted to the hot side and finding a close approximation of the integral. As α(T) may be quite different (see Figure A1b), we meet various situations, represented by different $\Delta T_{\mathrm{Thomson}}\left(x\right)$ temperature profiles (Figure A2b), among them typical ones with a single maximum according to Thomson heat flowing out to both sides, but also less typical ones with a single minimum (Thomson heat flowing in from both sides) or even two extrema (for Bi_2_Te_3_) where Thomson heat is released to the cold side but absorbed from the hot side. A rule to treat all of the cases likewise is needed. Figure 5a,b and Figure A2c,d accordingly show scenarios where α(T) contains almost linear intervals along with strongly bowed ones, where α(T) is monotonous or contains a maximum, where $\alpha_{h}$ and $\bar{\alpha }$ are far from each other or close together or where α(T) crosses the $\bar{\alpha }$ horizontal once or twice. The position of the extrema (maxima or minima) of $\Delta T_{\mathrm{Thomson}}\left(x\right)$ is marked in each diagram by a brown line. Accordingly, the area corresponding to the uncompensated heat might be more complex than is shown in Figure 3, e.g., see Figure 5a. The area to the left of the α(T) curve to the α-axis from this point up to the hot side $\alpha_{h}$ (marked by a red border) represents ${\dot{Q}}_{\tau ,h}$. The fact that the respective area also contains negatively counted parts when α(T) goes through a maximum is also taken into account. Accordingly, the upper slim boat-shaped area in Figure 5a counts as negative; symbolically, it is mirrored in the green area.

However, in such a case, the integration can be simplified, switching from the hot to the cold side, as ${\dot{Q}}_{\tau ,h}={\dot{Q}}_{\tau }-{\dot{Q}}_{\tau ,c}$ and with Equation (10), ${\dot{Q}}_{\tau }={\dot{Q}}_{\pi ,h}-{\dot{Q}}_{\pi ,c}-P_{0}$. Note that if there are two extrema of $\Delta T_{\mathrm{Thomson}}\left(x\right)$, then we have two $T_{\tau ,\mathrm{ex}}$ values where the Thomson heat between both can be neglected as it cancels out completely. Only the intervals outside, $\left(T_{c};T_{\tau ,\mathrm{ex}}\right)$ or $\left(T_{\tau ,\mathrm{ex}};T_{h}\right)$, have to be considered. Among both intervals, the side has to be chosen where α(T) is a monotonous function in the relevant temperature interval, where it is closer to linearity, and possibly where $T_{\tau ,\mathrm{ex}}$ is closer to $T_{h}$ or $T_{c}$.

Applying Equation (11) accordingly to the chosen interval, the integration for ${\dot{Q}}_{\tau }$ can be substituted by one for $P_{0}$, e.g., for the cold side:

(13) $${\dot{Q}}_{\tau ,c}={\dot{Q}}_{\pi ,T_{\tau ,\mathrm{ex}}}-{\dot{Q}}_{\pi ,c}-\int_{T_{c}}^{T_{\tau ,\mathrm{ex}}}\alpha dT$$

This facilitates practical execution as α(T) is mostly known as a low-order polynomial, thus integration could be done analytically.

If the Thomson T profile is not known, half of the leg length, $\frac{L}{2}$, can be taken as a first guess of the position for the calculation of ${\dot{Q}}_{\tau ,h}$. The corresponding temperature is marked in the diagrams. This can be a quite good estimate when the Thomson T profile is close to symmetric, as for PbTe (see Figure A2b), but may fail greatly when Thomson heat is strongly asymmetric, as for SnSe. On the contrary, an entropy consideration of Thomson heat in the TE leg (see Appendix A.4.) leads to a rule of thumb for $T_{\tau ,\mathrm{ex}}$ that is

(14) $$\alpha \left(T_{\tau ,\mathrm{ex}}\right)\approx \bar{\alpha }$$

Indeed, it applies well for all example materials involved here. With this rule, approximation of ${\dot{Q}}_{\tau ,h}$ is facilitated considerably, as just a crossing point of α(T) with its TAv has to be found.

Figure 6 shows the remaining efficiency deviation, $\delta \eta_{\max}^{\mathrm{corr}}$, corrected by the uncompensated Peltier–Thomson heat calculated from the α(T)  graph using the $\frac{L}{2}$ position, using $T_{\tau ,\mathrm{ex}}$ according to the extremum (maximum) position of $\Delta T_{\mathrm{Thomson}}\left(x\right)$ but neglecting the current deviation ${\delta I}_{\mathrm{opt},\eta }$, as well as corrected by the exact deviation ${d\dot{Q}}_{\pi \tau }^{h}={\dot{Q}}_{\pi ,h}-{\dot{Q}}_{\tau ,h}-{\dot{Q}}_{\pi ,h}{}^{\mathrm{CPM}}$. The efficiency estimate by the CPM is greatly improved when the $\Delta T_{\mathrm{Thomson}}\left(x\right)$ extremum position is used(red dots).

Only occasionally, e.g., when α(T) is close to linear, the $\frac{L}{2}$ position works well for correction but fails for most materials as it does not take into account the asymmetry of heat sources and heat conduction. Similarly, models suggesting half of the Thomson heat on either side for correcting the CPM results [14,15,16,26,27] will mostly not work sufficiently. The correction employing the $\Delta T_{\mathrm{Thomson}}\left(x\right)$ peak position is close to the exact numerical correction for most materials as this position considers the asymmetry exactly. The difference between both cases is merely due to the change of the optimum current which is as yet unconsidered by the graphical correction. The remaining discrepancy is due to Joule heat asymmetry.

Whereas we have used exact numerical calculations to demonstrate the principle of the Thomson correction method and to show that the rule $\alpha \left(T_{\tau ,\mathrm{ex}}\right)\approx \bar{\alpha }$ holds well, the suggested practical procedure for the correction of ${d\dot{Q}}_{\pi \tau }^{h,\;I=\mathrm{const}}$ described here, which is based on an analysis of the physical effects behind the deviation of CPM performance estimates, is limited to basic algebraic operations which can be instantaneously calculated by any table calculation software.

## 3. Conclusions

From the study of 2TD and 1TD model materials with one or two selected properties among α, ρ and κ set as constant, which results in both redistribution of heat between the hot and cold side of the element and the change of spatial averages, we see that in some examples, large deviations in efficiency $\delta \eta_{\max}^{\mathrm{model}}$ arise as a consequence of considerable modification of the *T* profile. In comparison to the efficiency deviation between the CPM and real materials ${\delta \eta }_{\max}$ which conserve the spatial property averages and are mostly below 2%, this shows that a change of spatial averages due to an arbitrary modification of the *T* profile may contribute a strong shift to the efficiency estimate. Thus, conservation of the leg’s thermal and electrical resistance is essential for a valid efficiency estimate. However, the shift mainly remains low if only ρ(*T*) is switched to constant. Nevertheless, it cannot be concluded from this that the temperature dependence of the electrical resistivity plays a minor role in the efficiency estimation by the CPM. The 2TD and 1TD model materials lead to quite good approximations of the partial *T* profiles $\Delta T_{\mathrm{Joule}}\left(x\right)$, $\Delta T_{\mathrm{Thomson}}\left(x\right)$ and $\Delta T_{\kappa \left(T\right)}\left(x\right)$.

It is shown that the deviation of a CPM-based efficiency estimate, ${\delta \eta }_{\max}$, is not just due to the absence of Thomson heat in the CPM, as the choice of the temperature average of α(T) as a CPM parameter mainly compensates for the absence of Thomson heat. Rather, the discrepancy in efficiency determination in the CPM is shown to be, to a major extent, due to the excess unaccounted heat at the hot side in the CPM ${\delta \dot{Q}}_{\mathrm{in}}$, which usually leads to overestimation of performance, and, to a minor extent, due to a shift of the optimum current ${\delta I}_{\mathrm{opt},\eta }$ and, consequently, of the produced electrical power at maximum efficiency, ${\delta P}_{\eta_{\max}}^{}$. In most cases, the change of the optimum current is small. In materials with rising α(T), less of the released Thomson heat flows back to the hot side than would compensate for the reduced hot side Peltier heat absorption assumed by the CPM. This systematic undercompensation tends towards a higher actual heat intake at the hot side compared to the CPM, thus overestimating efficiency when the CPM is used. Asymmetry of Joule heat usually has an opposite influence but is overcompensated in most cases.

In order to correct for the Peltier–Thomson heat-related deviation ${\delta \dot{Q}}_{\pi \tau }^{h}$, a graphical illustration in terms of convective entropy flow based on the α(T) curve is given. It confirms that the rule for the splitting of Thomson heat to the sides $\alpha \left(T_{\tau ,\mathrm{ex}}\right)\approx \bar{\alpha }$, which results from an entropy consideration, holds well. This enables a valid approximation of ${\delta \dot{Q}}_{\pi \tau }^{h}$ with a simple algebraic procedure, omitting the exact numerical calculation of the temperature profile. Although a considerable deformation of the T profile caused by the T dependence of κ(T) is observed, it will affect the deviation between the real situation and its CPM approximation simply via a local shift of the thermal and electrical resistivity but will not explicitly contribute to the inflowing heat balance ${\dot{Q}}_{\mathrm{in}}$.

In summary, the performance of a TE material does not only depend on its averaged material parameters but also on local asymmetry of Thomson and Joule heat, driven by the *T* dependence of the TE properties. In particular, Thomson heat can show highly asymmetric distribution. Thus, TE device efficiency can be varied beyond the averaged properties, represented by a figure of merit.

## Appendix A

### Appendix A.1. Note from Section 2.1

A very good approximation of the actual T profile and hence the SpAv of ρ and κ in accordance with a real material can be calculated in a straightforward way from the *T*-dependent properties without using an iterative solution [18] for T(x). This may considerably simplify the estimation of appropriate SpAvs as CPM property values. $\Delta T_{\mathrm{Joule}}\left(x\right)$ can be obtained analytically from the CPM case, $\Delta T_{\kappa \left(T\right)}$ from a integration of the Fourier equation and $\Delta T_{\mathrm{Thomson}}$ and from a 1TD α(T) model by a single integration.

### Appendix A.2. Material Data and Boundary Conditions

**Table A1:** Temperature range of analysis for all materials of Figure A1.

Material	Temperature Range of Analysis
p-Mg_2_(Si,Sn)	723 K to 383 K
n-Mg_2_(Si,Sn)	723 K to 383 K
HMS	833 K to 298 K
Mg_2_Si	833 K to 298 K
p-Bi_2_Te_3_	553 K to 301 K
SnSe	973 K to 373 K
PbTe	850 K to 320 K

### Appendix A.3. Additional Information

#### Appendix A.3.1. Finding Individual Contributions to the Total T Profile

The partial T profiles are each found by equating $\kappa \left(T\right)\frac{\partial^{2}T}{\partial x^{2}}$ in Equation (1) to each of the other corresponding terms, assuming isothermal boundary conditions and fixing all coefficients in the equation according to the total T profile T(x). Thus, solving for the respective partial T profile reduces to a double integration, where the first step provides the total amount of each partial heat contribution to the thermal balance.

As the partial T profiles can have opposite signs in amplitude and partially compensate for many of the common TE materials (however, not always), the T profiles of a real material and the CPM may be quite close, as in the example of *n*-type Mg_2_*X*, Figure 2b.

#### Appendix A.3.2. Contributions to ${\dot{Q}}_{\mathrm{in}}$

As both Joule and Thomson heat, after appearing inside the leg, will flow out, physically, as Fourier heat, we have to consider in this discussion the pure Fourier heat $Q_{F,h}=K\Delta T$ (with $K=\langle \kappa^{-1}\rangle {}^{-1}A/L$), which is merely related to the thermal resistance of the leg and is constant along the leg, separately from the Joule- and Thomson-related contributions. Accordingly, $Q_{\mathrm{in}}$ is composed of

(A1) $${\dot{Q}}_{\mathrm{in}}={\dot{Q}}_{F,h}+{\dot{Q}}_{\pi ,h}-{\dot{Q}}_{\tau ,h}-{\dot{Q}}_{J,h}$$

The real Joule- and Thomson-related contributions, $-{\dot{Q}}_{\tau ,h}$ and ${\dot{-Q}}_{J,h}$, to the inflowing hot side heat are calculated by splitting the overall temperature profile T(x) into additive partial T profiles, each related to one of the individual physical contributions. Partial Thomson *T* profiles of example materials are plotted in Figure A2b. Evaluating $-\kappa_{h}\cdot {\left(\frac{d}{dx}\Delta T_{\mathrm{Thomson}}\right)}_{h\;}$ and $-\kappa_{h}\cdot {\left(\frac{d}{dx}\Delta T_{\mathrm{Joule}}\right)}_{h\;}$ from the partial T profiles gives ${\dot{Q}}_{\tau ,h}$ and ${\dot{Q}}_{J,h}$, respectively.

Figure A2a shows the relative contribution of each heat to ${\dot{Q}}_{\mathrm{in}}$: $\frac{{\dot{Q}}_{F,h}}{{\dot{Q}}_{\mathrm{in}}},\frac{{\dot{Q}}_{\pi ,h}}{{\dot{Q}}_{\mathrm{in}}},-\frac{{\dot{Q}}_{J,h}}{{\dot{Q}}_{\mathrm{in}}},-\frac{{\dot{Q}}_{\tau ,h}}{{\dot{Q}}_{\mathrm{in}}}$. This comparison reveals that Joule and Thomson heat contribute about 1–5% to ${\dot{Q}}_{\mathrm{in}},$ usually flowing out, with their contributions being roughly of the same order. Figure A2a also shows the fraction of Thomson heat and Joule heat distributed to the hot side ($\frac{{\dot{Q}}_{\tau ,h}}{{\dot{Q}}_{\tau }}$ and $\frac{{\dot{Q}}_{J,h}}{{\dot{Q}}_{J}}$).

In order to illustrate example situations of the distribution of Peltier and Thomson heat along the leg, α(T) graphs for p-Mg_2_*X* and Bi**_2_**Te_3_ are given in Figure A2c,d, respectively. Due to the bowed shape of the α(T) graph and relatively close values of $\alpha_{h}$ to $\alpha_{c}$ for p-Mg_2_*X*, the difference between ${\dot{Q}}_{\pi ,h}$ and ${\dot{Q}}_{\pi ,h}^{\mathrm{CPM}}$ is almost negligible, but ${\dot{Q}}_{\tau ,h}$ amounts to more than twice the amount of ${\dot{Q}}_{\pi ,h}-{\dot{Q}}_{\pi ,h}^{\mathrm{CPM}}$. Nevertheless, this did not affect the efficiency deviation ${\delta \eta }_{\max}$ too much, as ${\dot{Q}}_{\tau ,h}$ is quite small in absolute terms. In the case of Bi_2_Te_3_, ${\dot{Q}}_{\pi ,h}^{\mathrm{CPM}}$ is even higher than ${\dot{Q}}_{\pi ,h}$ again due to the curved shape of α(T), affecting the position of $\alpha_{\mathrm{TAv}}$. However, ${\dot{Q}}_{\tau ,h}$ almost completely compensates for this Peltier heat difference, keeping the influence on the efficiency deviation negligible.

### Appendix A.4. Thomson Heat Distribution and Entropy

With the TEG leg, we discuss the entropy flow in a reversible system of Peltier heat transport and Thomson heat exchange which is running on a non-equilibrium temperature background mainly fixed by the continuous flow of Fourier heat. As released Thomson heat will be transported as Fourier heat but is small in relation to the Fourier heat background (see Figure A2a), which is driven by the temperature difference and the thermal resistance of the TE leg, we will treat the variation of the temperature profile by the conducted Thomson heat as insignificant for the following consideration.

In the steady state, the entropy of the system remains constant; there is a continuous entropy production by the dissipative heat transport from hot to cold and the balancing continuous entropy export by transmitted Fourier heat (plus a negligible fraction arising from outflowing Joule heat). Assuming ideal outer current leads with α=0, there is no other entropy exchange at the hot and cold sides.

In the CPM, we have a constant convective entropy flow $\bar{\alpha }I$ throughout the element, equal to the absorbed and released entropy rate $\bar{\alpha }I$ by absorption and release of Peltier heat at the terminals. In a real material, the absorbed entropy rate $\alpha_{h}I$ equals the convective entropy flow at the hot side, and, likewise, the amount of $\alpha_{c}I$ at the cold side. The variation of α along the leg drives local Thomson heat production $d{\dot{Q}}_{\tau }=T\frac{d\alpha }{dT}IdT=TId\alpha$, contributing an entropy flow increment $d\dot{S}=Id\alpha$. Thomson heat flows to the hot and cold sides and the related total entropy exchange is $\left(\alpha_{h}-\alpha_{c}\right)I$ = ΔαI. It distributes by the fraction $x_{h}$ to the hot and cold sides:

(A2) $$\Delta {\dot{S}}_{\tau ,h}=x_{h}\left(\alpha_{h}-\alpha_{c}\right)I\;\mathrm{and}\;\Delta {\dot{S}}_{\tau ,c}=\left(1-x_{h}\right)\left(\alpha_{h}-\alpha_{c}\right)I.$$

Driven by the gradient of the partial Thomson temperature profile, all Thomson heat released at one side of a maximum (or minimum) of this profile will be exchanged to this side of the leg. With the temperature $T_{\tau ,\mathrm{ex}}$ of this position and its Seebeck coefficient $\alpha_{\tau ,\mathrm{ex}}=\alpha \left(T_{\tau ,\mathrm{ex}}\right)$, the shares of the entropy exchange which are bound to each of the sides are

(A3) $$\Delta {\dot{S}}_{\tau ,h}=\left(\alpha_{h}-\alpha_{\tau ,\mathrm{ex}}\right)I\;\mathrm{and}\;\Delta {\dot{S}}_{\tau ,c}=\left(\alpha_{\tau ,\mathrm{ex}}-\alpha_{c}\right)I.$$

Multiplying both by the respective temperature of the side yields total Thomson heat:

(A4) $$\begin{array}{c} {\dot{Q}}_{\tau }=T_{h}\left(\alpha_{h}-\alpha_{\tau ,\mathrm{ex}}\right)I+T_{c}\left(\alpha_{\tau ,\mathrm{ex}}-\alpha_{c}\right)I=\left\{T_{h}\alpha_{h}-T_{c}\alpha_{c}-\alpha_{\tau ,\mathrm{ex}}\left(T_{h}-T_{c}\right)\right\}I=\Delta {\dot{Q}}_{\pi }-\\ I\alpha_{\tau ,\mathrm{ex}}\Delta T. \end{array}$$

Comparing Equation (A4) with the energy balance of reversible heat ${\dot{Q}}_{\tau }=\Delta {\dot{Q}}_{\pi }-IV_{0}$, we can conclude that

(A5) $$\alpha_{\tau ,\mathrm{ex}}\Delta T=V_{0}=\bar{\alpha }\Delta T,\;\mathrm{thus}\;\alpha_{\tau ,\mathrm{ex}}=\bar{\alpha }$$

This gives us a rule for the temperature intervals over which the Thomson heat is flowing to either side of the leg. Consequently, Thomson heat has to be integrated from the crossing point of the curve of the Seebeck coefficient α(T) with its temperature average $\bar{\alpha }$. As a reversible approximation, this result is approximate and not strict as we have neglected here that dissipative processes are involved when Thomson heat is conducted to the leg sides. Below we will analyze these changes and find that these are small, and thus the rule stated here on the position of $\alpha_{\tau ,\mathrm{ex}}$, although not strict, is a good guide for estimates of the distribution of Thomson heat. Indeed, as observed by comparison to exact numerical calculations, this rule is almost perfectly fulfilled for all the example materials.

Within this reversible approximation, the Thomson heat flowing to the hot side is obtained as ${\dot{Q}}_{\tau ,h}=T_{h}\left(\alpha_{h}-\bar{\alpha }\right)I$. This would be equivalent to a complete compensation of the Peltier heat difference between reality and the CPM, i.e., the vanishing axial redistribution of reversible heat which is consistent with the simplifying assumption that the Thomson heat flowing to the outside is transmitted free of dissipation, i.e., equivalent to reversible heat. Here, the (additional) *T* gradient related to the flow of Thomson heat is neglected, whereas an underlying *T* profile related to an independent heat flow (here, the background of Fourier heat transfer) does, in effect, not contribute to its dissipation. We will see below that this happens as Thomson heat flowing to different sides will contribute almost compensating shares to the entropy balance. What is neglected here is that the Thomson heat itself when flowing to the ends of the leg will dissipate, according to the slight shift of the inner *T* profile it is causing. Above, this *T* offset was separated and called the partial *T* profile due to Thomson heat, $\Delta T_{\mathrm{Thomson}}\left(x\right)$. Additionally, this omission will contribute to a weak deviation from the position rule $\alpha_{\tau ,\mathrm{ex}}=\bar{\alpha }$.

The dissipative part of the entropy transport to the sides of the leg is related to the *T* drop or step-up between the location where an increment of Thomson heat $d{\dot{Q}}_{\tau }$ is released and the side temperature, $T_{h}$ or $T_{c}$. The entropy increment is released over a segment of the leg with the *T* increment dT is $d\dot{S}=Id\alpha =\frac{d{\dot{Q}}_{\tau }}{T}$. With the transfer to the cold side, for example, the transmitted increment of Thomson heat $d{\dot{Q}}_{\tau }$ increases its entropy up to $d\dot{S_{c}}=\frac{d{\dot{Q}}_{\tau }}{T_{c}}$, and the according entropy gain is

(A6) $$d\Delta \dot{S_{c}}=d\dot{S_{c}}-d\dot{S}=\frac{d{\dot{Q}}_{\tau }}{T_{c}}-\frac{d{\dot{Q}}_{\tau }}{T}=\frac{d{\dot{Q}}_{\tau }}{TT_{c}}\left(T-T_{c}\right)=d\dot{S}\frac{T-T_{c}}{T_{c}}$$

Summing over all Thomson heat flowing to that side, we have

(A7) $$\Delta \dot{S_{c}}=\frac{1}{T_{c}}\int_{I\alpha_{c}}^{\bar{I\alpha }}\left(T-T_{c}\right)d\dot{S}=\frac{I}{T_{c}}\int_{\alpha_{c}}^{\bar{\alpha }}\left(T-T_{c}\right)d\alpha =\frac{I}{T_{c}}\int_{T_{c}}^{T_{\bar{\alpha }}}T\frac{d\alpha }{dT}dT-I\left(\bar{\alpha }-\alpha_{c}\right)$$

Multiplying with the cold side temperature, $\Delta {\dot{Q}}_{\tau ,c}=T_{c}\Delta \dot{S_{c}}=\int_{T_{c}}^{T_{\bar{\alpha }}}T\frac{d\alpha }{dT}dT-I\left(\bar{\alpha }-\alpha_{c}\right)T_{c}$ gives us the amount of Thomson heat that is just the difference from the Peltier–Thomson heat balance of the CPM, $\Delta {\dot{Q}}_{\tau ,c}={\dot{Q}}_{\tau ,c}-\left({\dot{Q}}_{\pi ,c}^{\mathrm{CPM}}-{\dot{Q}}_{\pi ,c}\right)$, i.e., the part that we have identified as uncompensated Peltier–Thomson heat in a real material. Note that ${\dot{Q}}_{\pi ,c}^{\mathrm{CPM}}$ contains merely completely reversible exchange of Peltier heat. Thus, the incomplete compensation of the Peltier–Thomson heat balance can be understood as an effect of the partly dissipative character of the exchange of the Thomson heat in a real system when conducted to the side. Accordingly, with the same consideration for the hot side, with $d\dot{S_{h}}=\frac{d{\dot{Q}}_{\tau }}{T_{h}}$, we obtain

(A8) $$\Delta \dot{S_{h}}=d\dot{S_{h}}-d\dot{S}=\frac{d{\dot{Q}}_{\tau }}{T_{h}}-\frac{d{\dot{Q}}_{\tau }}{T}=\frac{I}{T_{h}}\int_{\bar{\alpha }}^{\alpha_{h}}\left(T-T_{h}\right)d\alpha =\frac{I}{T_{h}}\int_{T_{\bar{\alpha }}}^{T_{h}}T\frac{d\alpha }{dT}dT-I\left(\alpha_{h}-\bar{\alpha }\right)$$

i.e., $\Delta \dot{S_{h}}$ gives a negative contribution to the entropy balance. This sounds contradictory to the second law of thermodynamics but it is not, as the Thomson heat is not really flowing from a lower to a higher temperature but, when released, reduces the *T* gradient of the underlying background of flowing Fourier heat, thus reducing the Fourier heat flow by the amount of “upstreaming” Thomson heat.

The hot and cold side entropy changes together give

(A9) $$\begin{array}{c} \Delta \dot{S}=\Delta \dot{S_{h}}+\Delta \dot{S_{c}}=\frac{I}{T_{h}}\int_{\bar{\alpha }}^{\alpha_{h}}\left(T-T_{h}\right)d\alpha +\frac{I}{T_{c}}\int_{\alpha_{c}}^{\bar{\alpha }}\left(T-T_{c}\right)d\alpha =\frac{I}{T_{c}}\int_{T_{c}}^{T_{\bar{\alpha }}}T\frac{d\alpha }{dT}dT+\\ \frac{I}{T_{h}}\int_{T_{\bar{\alpha }}}^{T_{h}}T\frac{d\alpha }{dT}dT-I\left(\alpha_{h}-\alpha_{c}\right). \end{array}$$

With $\frac{1}{T_{c}}\int_{\alpha_{c}}^{\bar{\alpha }}\;Td\alpha \tilde{>}\bar{\alpha }-\alpha_{c}$ and $\frac{1}{T_{h}}\int_{\bar{\alpha }}^{\alpha_{h}}Td\alpha \tilde{<}\alpha_{h}-\bar{\alpha }$ we get $\frac{I}{T_{c}}\int_{T_{c}}^{T_{\bar{\alpha }}}T\frac{d\alpha }{dT}dT+\frac{I}{T_{h}}\int_{T_{\bar{\alpha }}}^{T_{h}}T\frac{d\alpha }{dT}dT\approx$$I\left(\alpha_{h}-\alpha_{c}\right)$ and thus $\Delta \dot{S}\approx 0$. Hence, assuming $\alpha_{\tau ,\mathrm{ex}}=\bar{\alpha }$, the entropy balance of the inner Thomson heat transfer as an offset of a much larger background Fourier heat flow is almost zero. This indeed confirms our approach to deduce a rule for the local distribution of Thomson heat based on a reversible approximation, i.e., assuming $\Delta \dot{S}\approx 0$, but also shows that the rule is not completely strict.
